# Urticaria after breakthrough Omicron BA.5.1 severe acute respiratory syndrome coronavirus 2 infection in a triple-vaccinated (Pfizer) patient: a case report

**DOI:** 10.1186/s13256-023-03904-2

**Published:** 2023-05-04

**Authors:** Karl Ciuoderis, Laura Perez, Catalina Alvarez, Jaime Usuga, Leidi Carvajal, Andrés Cardona, Maria A. Maya, Gavin Cloherty, Juan P. Hernandez-Ortiz, Jorge E. Osorio

**Affiliations:** 1 Laboratorio Genómico One Health, UW-GHI One Health Colombia, Universidad Nacional de Colombia, Cll 75#79A-51, Bloque M15, 050034 Medellin, Colombia; 2 Division of Infectious Diseases, San Vicente Fundación Hospital, Medellín, Colombia; 3 Abbott Diagnostics, Abbott Park, Chicago, USA; 4 Global Health Institute, University of Wisconsin-Madison, Madison, USA

**Keywords:** Urticaria, Skin, Rash, SARS-CoV-2, Postvaccination, Case report

## Abstract

**Background:**

Severe acute respiratory syndrome coronavirus 2 continues to threaten public health. The virus is causing breakthrough infections in vaccinated individuals. Also, scarce information is available about cutaneous manifestations after severe acute respiratory syndrome coronavirus 2 infection.

**Case presentation and findings:**

A case of a triple-vaccinated (Pfizer) 37-year-old Hispanic American (Colombian) male who developed urticaria after Omicron BA.5.1 severe acute respiratory syndrome coronavirus 2 breakthrough infection is described. Virus isolation and whole genome sequencing along with immune and molecular assays were performed. Dermatological manifestations (skin rash and urticaria) after Omicron BA.5.1 infection were observed. Sequence analysis of the Omicron BA.5.1 isolate also revealed several important mutations. Hemogram analysis revealed leukocytosis and neutrophilia. Serology testing revealed anti-spike immunoglobulin G serum titers but negative detection of immunoglobulin M at 10 days after symptom onset. Anti-nucleocapsid, anti-spike 1 immunoglobulin G, anti-spike trimer, and anti-receptor-binding-domain immunoglobulin G and immunoglobulin E sera were detected at different titers 10 days after symptom onset. Several serum levels of chemokines/cytokines (Interferon-α, interferon-γ, interleukin-12/interleukin-23p40, interleukin-18, interferon gamma-induced protein-10, monocyte chemoattractant protein-1, monokine induced by gamma, macrophage inflammatory protein-1α, chemokine (C-C motif) ligand-5 , tumor necrosis factor-β1, Tumor necrosis factor-α) were detected, but interleukin-2, interleukin-4, interleukin-6, interleukin-8, and interleukin-17A were below the limit of detection.

**Interpretation and conclusions:**

To our knowledge, this is the first study describing skin effects of a severe acute respiratory syndrome coronavirus 2 Omicron BA.5 variant breakthrough infection in a triple-vaccinated patient in Colombia. Several important mutations were found in the spike glycoprotein of the virus isolated; these mutations are associated with immune evasion and changes in antigenic properties of the virus. Physicians overseeing coronavirus disease 2019 cases should be aware of the potential skin effects of the infection. Pathogenesis of severe acute respiratory syndrome coronavirus 2 infection and its association with proinflammatory cytokines and chemokines may enhance the development of urticaria and other skin manifestations in immunized individuals. However, further studies are needed to better understand the complexity of coronavirus disease in such situations.

**Supplementary Information:**

The online version contains supplementary material available at 10.1186/s13256-023-03904-2.

## Background

Coronavirus disease 2019 (COVID-19) caused by severe acute respiratory syndrome coronavirus 2 (SARS-CoV-2) currently continues as a global problem even after vaccination strategies have been implemented. Virus evolution is causing breakthrough infections in vaccinated individuals [1]. Although it is now clear that SARS-CoV-2 can affect several organs, less is known about its cutaneous effects. Dermatological manifestations associated with COVID-19 have been increasingly reported from different geographical regions [2]. In this report, we describe a case of a triple-vaccinated (Pfizer) patient who developed urticaria after Omicron BA.5.1 SARS-CoV-2 infection. To date, this is one of the very few cases in which the immune humoral and cellular response to COVID-19 Omicron variant breakthrough infection has been investigated coupled to virus genome analysis to better characterize the disease findings.

## Case presentation and investigations

**Fig. 1:**
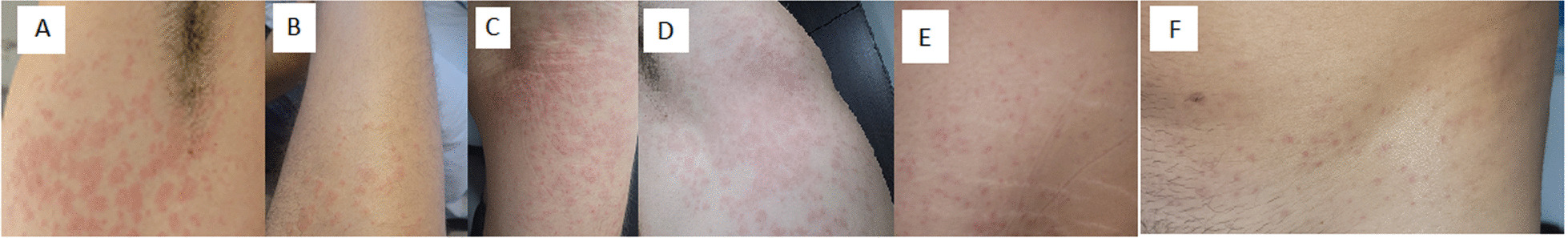
Clinical spectrum of the urticaria. **A** shows the rash on the left part of the trunk; **B** shows the rash on the left leg; **C** shows rash progression on the leg 1 day after the skin manisfestation’s onset; **D** shows rash progression on the trunk 1 day after; and **E** and **F** show the beginning of the rash in other body areas 3 days after the skin manisfestation’s onset

A 37-year-old male Hispanic American (Colombian) patient reported several symptoms of COVID-19, including persistent fever, fatigue, myalgia, severe headache, runny nose, odynophagia, and cough, on 19 June 2022. The patient was triple vaccinated (Pfizer), receiving the last dose on 16 February 2022. The patient was diagnosed with COVID-19 infection by both rapid diagnostic antigen test and reverse transcription polymerase chain reaction (RT-PCR) 2 days after symptom onset. At the time of diagnosis, the patient did not report any infection-related skin manifestations, immunosuppression history, or known COVID-19 comorbidities or chronic diseases. The patient did not use any medication regularly and had no history of exposure to a contact allergen. Also, the patient reported a previous infection (May 2021; RT-PCR confirmed). Within a week after symptom onset, respiratory disease progression was moderate to mild (headache, congestion, cough, runny nose, and odynophagia), but no hospitalization or intensive care was required. After self-care, recovery from respiratory illness was achieved within a week, but 7 days after symptom onset, the patient reported a mild rash and skin lesions localized on the lateral and medial regions of the body. The skin rash—raised, well-circumscribed, with erythema and edema, very pruritic (Fig. 1A, B)—progressed from localized exanthem to purpuric lesions (Fig. 1C, D).

Two days after the urticaria began, it extended to other body areas (Fig. 1E, F). Intense pruritus, burning, and pain in the affected skin areas were the main symptoms reported. Initial treatment with a combination of topical corticoids (0.1% betamethasone) and oral antihistamines (5 mg desloratadine and 180 mg fexofenadine) was unsuccessful. The patient received a single intramuscular dose of 4 mg dexamethasone on the fifth day after symptom onset. Symptoms were relieved 24 hours after corticosteroid injection, and 100% clinical resolution was reported 20 days after symptom onset. Hemogram analysis (24 hours after corticosteroid injection) revealed leukocytosis and neutrophilia (Table 1).

**Table 1:** Routine automated blood test (hemogram) results at 5 days after symptom onset and normal reference values

Variable	Value	Reference range	Variable	Value	Reference range
Total white blood cells	10.59 × 10^9^/L	4.0–10.0 × 10^9^/L	Red blood cells	5.51 × 10^6^/L	4.1–6.1 × 10^6^/L
Neutrophils	8.18 × 10^9^/L (77.2%)	2–7.5 × 10^9^/L	Platelets	380 × 10^9^/L	150–450 × 10^9^/L
Lymphocytes	2.00 × 10^9^/L (18.9%)	1.5–4.5 × 10^9^/L	Hemoglobin	15.3 g/dL	13–18 g/dL
Monocytes	0.37 × 10^9^/L (3.5%)	0.2–0.8 × 10^9^/L	Hematocrit	47.9%	40–52%
Eosinophils	0.02 × 10^9^/L (0.2%)	0.0–0.4 × 10^9^/L	Mean corpuscular volume	86.9 fL	80–100 fL
Basophils	0.02 × 10^9^/L (0.2%)	0.0–0.1 × 10^9^/L	Mean corpuscular hemoglobin concentration	31.9 g/dL	32–36 g/dL

Serology testing by chemiluminescence assay revealed total anti-spike (S) immunoglobulin G (IgG) serum titers of 15,362 AU/mL and negative detection of immunoglobulin M (IgM; index of 0.57) on the 10th day after symptom onset. Anti-nucleocapsid IgG, anti-spike-1 (S1) IgG, anti-S-trimer IgG, and anti-receptor-binding-domain (RBD) IgG serum titers were 1232 U/mL, 246 U/mL, 226 U/mL, and 334 U/mL, respectively. Total serum immunoglobulin E (IgE) level at 10 days after symptom onset was 7.0 IU/mL. Ten days after symptoms onset (24 h after corticoid injection), the patient had serum levels of 1.02 pg/mL of interferon alpha (IFNα), 3.03 pg/mL of interferon gamma (IFNγ), 0.74 pg/mL of interleukin (IL)-12/IL-23p40, 3.43 pg/mL of IL-18, 21.04 pg/mL of interferon-gamma-induced protein (IP)-10, 19.78 pg/mL of monocyte chemoattractant protein (MCP)-1, 3.1 pg/mL of monokine induced by gamma (MIG), 6.9 pg/mL of macrophage inflammatory protein-1 alpha (MIP-1α), 22.8 pg/mL of chemokine (C-C motif) ligand 5 (CCL5), 4.6 pg/mL of tumor necrosis factor beta-1 (TNFβ1), and 2.0 pg/mL of tumor necrosis factor alpha (TNFα). Serum levels of IL-2, IL-4, IL-6, IL-8, and IL-17A were below the limit of detection. The limit of detection (multiplex immunoassay) for serum cytokines and chemokines ranged from 0.62 to 2.431 pg/mL (standard controls).

SARS-CoV-2 was detected by RT-PCR [cycle threshold (Ct) value of 24.1; 30,225 RNA copies] on a nasal swab collected 11 days after symptom onset. After whole genome sequencing and virus sequence analysis, Omicron BA.5.1 lineage was identified. Sequencing data are available at the Global Initiative on Sharing Avian Influenza Data (GISAID) repository (Accession N° EPI_ISL_13822391). Sequence analysis of the Omicron BA.5.1 isolate also revealed several genomic mutations. Compared with reference BA.2, the Omicron BA.5.1 isolate had 75 mutations.

The most frequent events were single nucleotide polymorphisms in the spike (S) protein (43.1%;31/72). Four important mutations (K417N, T478K, E484A, and N501Y) were found in the S-glycoprotein; these mutations are known in the Omicron variant for being associated with destabilization of the antibody-binding affinity [3]. Bioinformatics analysis also revealed that some mutations in the open reading frame, S, and matrix proteins had moderate impact (substitutions that produced a different amino acid from the usual amino acid at that position) and thus may alter the function of the proteins. Compared with reference human coronavirus 2019 (hCoV-19)/Wuhan/WIV04/2019, the Omicron BA.5.1 isolate revealed 28 mutations in the S protein, which are related to predicted biological effects and epidemiological significance in the antigenic properties of the virus. Most mutations are involved in altering host-cell receptor binding or antigenicity. However, three mutations (L24del, P25del, and P26del) were related to deletion of amino acid residues, and one mutation (T19I) affected a potential glycosylation site (Table 2). Detailed description of virus genomic sequence and mutations found in this report are provided (Additional file 1).

**Table 2:** Analysis of mutations in the spike protein of the Omicron BA.5.1 SARS-CoV-2 using several bioinformatic tools

Mutations identified	Description of biological significance
T19I	Amino acid changes that create or remove a potential glycosylation site
L24del, P25del, P26del	Amino acid changes that lead to a deletion of amino acid residues
A27S, G142D, V213G, S375F, T376A, H655Y, N679K, N764K, D796Y, Q954H, N969K	Amino acid changes occurring more than 100 times that are more interesting epidemiologically
H69del, V70del, G339D, S371F, S373P, D405N, R408S, K417N, N440K, L452R, S477N, T478K, E484A, F486V, Q498R, N501Y, Y505H, D614G, P681H	Amino acid changes that occur at a site known to be involved in phenotypic effects such as altering host-cell receptor binding or antigenicity
Reference: hCoV-19/Wuhan/WIV04/2019 (Wuhan)	

Molecular testing, virus sequencing, and phylogenetic analysis were conducted following protocols reported elsewhere [4, 5]. Additionally, antibodies (IgG anti-spike, anti-RBD, and anti-Nucleocapsid SARS-CoV-2) and serum cytokine/chemokine (18 analytes) were detected using ProcartaPlex multiplex immunoassays (Thermo Fisher Scientific, MA, USA) for the Luminex xMAP instrument (Luminex Corporation, TX, USA) following the manufacturer’s instructions. Serology testing was also performed on the Abbott ARCHITECT i1000 instrument using the Abbott SARS-CoV-2 quantitative IgG and qualitative IgM assays (Abbott Park, IL, USA). Virus genome sequence analysis was conducted using Pangolin and CoronApp tools [6, 7]. Additional bioinformatics analysis of virus mutations was conducted using the MicroGMT tool [8] and CoVsurver/GISAID [9]. A routine automated blood test (hemogram) was obtained by the standard method. Virus isolation was performed as previously described [10].

We conducted a subsequent review of the literature to search for cases of patients who presented skin manifestations after vaccination or infection. We referred to the electronic databases (PubMed) using the terms “COVID-19” or “SARS-CoV-2” in combination with “skin” or “cutaneous manifestations” or “rash” to see if any other cases of skin manifestations had been reported. Deidentified case study data as well as an informed consent form are available upon request after signing a data access agreement.

## Discussion and conclusions

To our knowledge this is the first study describing skin effects of a SARS-CoV-2 Omicron BA.5 variant breakthrough infection in a triple-vaccinated patient in Colombia. Since the advent of vaccines to prevent SARS-CoV-2 infection, there has been a great reduction in infections worldwide, but reports of COVID-19 breakthrough infections in vaccinated individuals are increasing [11]. Some studies have shown infection due to extensive escape of the SARS-CoV-2 Omicron variant in vaccinated individuals [12]. Several symptoms of SARS-CoV-2 infection in vaccinated individuals have been reported so far, including some “long COVID-19” symptoms [13]; however, reports of skin manifestations are rare. Some COVID-19 studies have reported cutaneous symptoms [14], and it seems that these cutaneous effects of SARS-CoV-2 infection may be similar to those of other common viral infections [15].

The immune response to SARS-CoV-2 infection can be associated with localized morbilliform rash, petechial rash, erythematous-to-purpuric coalescing macules, and urticaria [16]. Cutaneous manifestations associated with COVID-19 in our patient suggest the activation of pathogenic pathways by the virus or as a result of the inflammatory response. The pathogenesis of urticaria is immunologically related to mast cell (MC) degranulation. Mucosal tissues, as well as the skin, are body areas rich in MC. Therefore, we suggest that the urticaria, limited to specific body regions, was caused by the immune response to viral infection, and may not be related to a drug or treatment reaction or other causes.

Urticarial dermatosis after COVID-19 vaccination with unsuccessful antihistamine treatment and similar skin manifestations to those described in our patient has been reported [17]. Urticaria reaction may be associated with an intense inflammatory response and marked upregulation of some MC-derived cytokines [18]; however, pathogenesis of urticaria associated with COVID-19 has yet to be elucidated. Studies have shown that SARS-CoV-2 infection triggers MC degranulation, which causes hyperinflammation and injury [19]. Pathogen-associated molecular patterns can activate MC during SARS-CoV-2 infection, inducing an extremely high level of proinflammatory cytokines and chemokines [19], and also eliciting an influx, activation, and recruitment of neutrophils [17]. Moreover, MC communicate with endothelial cells, fibroblasts, and macrophages, further stimulating release of proinflammatory and vasoactive mediators [20]. Other viral infections such as dengue have been associated with similar MC degranulation mechanisms [21]. Also, retrospective analyses have shown that exacerbation of chronic spontaneous urticaria are associated with COVID-19 [22]. However, relationships and mechanisms of SARS-CoV-2 infection, its pathogenesis, and cutaneous effects are worthy of further investigation.

Studies showed that effects of COVID-19 as well as vaccines can induce antibody-dependent enhancement (ADE) reactions [23]. A study demonstrated that SARS-CoV-2 infection produces humoral response that elicits ADE, but these antibodies do not contribute to excess cytokine production by macrophages [23]. In addition, the binding of IgE and IgG antibodies to multivalent antigens may trigger MC degranulation, which would initiate allergic and inflammatory reactions and serve as a chemoattractant for other immune cells [24]. Also, maternally acquired SARS-CoV-2 antibodies bound to MC are possible causes of multisystem inflammatory reactions in children [25]. Thus, subneutralizing or cross-reactive non-neutralizing antiviral antibodies after vaccination or natural infection can contribute to subsequent ADE reactions. Therefore, we hypothesized that anti-SARS-CoV-2 IgG antibody levels, in addition to potential IgE antibodies induced by the recent Omicron infection and past vaccination in our patient, exacerbated inflammatory reactions and induced MC degranulation via ADE. This potential model increased emphasis on the importance of developing SARS-CoV-2 vaccines that are not dependent only upon antibody protection.

Less is known regarding Omicron-induced cellular and humoral responses after a breakthrough infection in vaccinated individuals with and without prior infection. Anti-spike IgG titers observed in our patient were about half of the median antibody titers (25,468 AU/mL) reported by other studies in triple-vaccinated adults 7 months after the first dose [26]. Interestingly, the patient did not show anti-SARS-CoV-2 IgM titers 10 days after symptom onset, but virus shedding titers were still high (Ct value 24.1). Also, anti-S1, S-trimer, and RBD were low compared with those reported in other studies in triple-vaccinated individuals [27]. We observed decreased serum levels of cytokines and chemokines compared with levels on healthy controls measured in other COVID-19 studies [28]. The overall dampening effect on cytokine and chemokine concentrations may be associated with dexamethasone treatment, as reported in other studies [29]. This suggest that previous SARS-CoV-2 infection, as well as low preinfection IgG antibody levels as observed in our patient, might impact Omicron-induced responses in triple-vaccinated individuals, but further investigations are needed.

The sequence analysis of the virus associated with our triple-vaccinated patient’s Omicron BA.5.1 infection revealed a high number of mutations in this virus, particularly in the spike protein, which confirms the fears about the Omicron variant’s capacity to resist pre-existing immunity acquired by vaccination or natural infection [30]. The amino acid substitutions found in the virus may alter the functions of the proteins in such a way that the molecular mechanisms could be associated with enhancement of pathogenesis, systemic hypersensitivity, and exacerbated inflammatory reactions causing the urticaria; however, further investigation is needed to better understand these mechanisms.

With the introduction of Delta and Omicron variants into Colombia, and the current evolution of the virus, additional studies are needed to better understand the impact of the current SARS-CoV-2 variants in fully vaccinated populations. The recent introduction of the SARS-CoV-2 BA.5 variant in Colombia has produced an increase in COVID-19 cases in which breakthrough infections are reported. We do not know if the immune status of the population would induce or prevent cutaneous manifestations in these breakthrough infections, therefore highlighting the need of more extensive studies. However, the findings provided in this work are key for physicians overseeing COVID-19 cases when it comes to considering potential skin effects of SARS-CoV-2 infections.

## Supplementary Information

**Additional file 1**. Results of mutations analysis of the Omicron BA.5.1 SARS-COV-2 isolate from this study.

## Data Availability

The authors confirm that the data supporting the findings of this study are available within the article and its supplementary materials. Raw data and other additional information are available from the corresponding author upon request.

## Abbreviations

SARS-CoV-2: Severe acute respiratory syndrome coronavirus 2
IgG: Immunoglobulin G
IgM: Immunoglobulin M
S: Spike protein
COVID-19: Coronavirus disease 2019

## References

[1] Coburn SB, Humes E, Lang R, Stewart C, Hogan BC, Gebo KA (2022). Analysis of postvaccination breakthrough COVID-19 infections among adults with HIV in the United States. JAMA Netw Open.

[2] Sodeifian F, Mushtaq S, Rezaei N (2022). Cutaneous manifestation of COVID-19: what have we learned an year into the pandemic?. Actas Dermosifiliogr.

[3] Bhattacharya M, Sharma AR, Dhama K, Agoramoorthy G, Chakraborty C (2022). Omicron variant (B.1.1.529) of SARS-CoV-2: understanding mutations in the genome, S-glycoprotein, and antibody-binding regions. GeroScience..

[4] Quick J. nCoV-2019 sequencing protocol 2020. 2020. https://www.protocols.io/view/ncov-2019-sequencing-protocol-bbmuik6w.

[5] Corman V, Bleicker T, Brünink S, Drosten C, Landt O, Koopmans M, *et al*. Diagnostic detection of 2019-nCoV by real-time RT-PCR. World Health Organization. 2020. https://www.who.int/docs/default-source/coronaviruse/protocol-v2-1.pdf.

[6] O’Toole Á, Scher E, Underwood A, Jackson B, Hill V, McCrone JT (2021). Assignment of epidemiological lineages in an emerging pandemic using the Pangolin tool. Virus Evol..

[7] Mercatelli D, Triboli L, Fornasari E, Ray F, Giorgi FM (2021). Coronapp: a web application to annotate and monitor SARS-CoV-2 mutations. J Med Virol.

[8] Xing Y, Li X, Gao X, Dong Q (2020). MicroGMT: a mutation tracker for SARS-CoV-2 and other microbial genome sequences. Front Microbiol.

[9] Khare S, Gurry C, Freitas L, Schultz MB, Bach G, Diallo A (2021). GISAID’s role in pandemic response. China CDC Wkly.

[10] Yao P, Zhang Y, Sun Y, Gu Y, Xu F, Su B (2020). Isolation and growth characteristics of SARS-CoV-2 in Vero cell. Virol Sin.

[11] Zhang M, Liang Y, Yu D, Du B, Cheng W, Li L (2022). A systematic review of vaccine breakthrough infections by SARS-CoV-2 Delta variant. Int J Biol Sci.

[12] Khan K, Karim F, Cele S, San JE, Lustig G, Tegally H, *et al*. Omicron infection of vaccinated individuals enhances neutralizing immunity against the Delta variant. MedRxiv Prepr Serv Health Sci. [Preprint]. 2022:2021.12.27.21268439. 10.1101/2021.12.27.21268439. Update in: Nature. 2022;602(7898):654–6. 10.1038/s41586-021-04387-1.

[13] Bergwerk M, Gonen T, Lustig Y, Amit S, Lipsitch M, Cohen C (2021). Covid-19 breakthrough infections in vaccinated health care workers. N Engl J Med.

[14] Criado PR, Abdalla BMZ, de Assis IC, van Blarcum de Graaff MC, Caputo GC, Vieira IC (2020). Are the cutaneous manifestations during or due to SARS-CoV-2 infection/COVID-19 frequent or not? Revision of possible pathophysiologic mechanisms. Inflamm Res.

[15] Morey-Olivé M, Espiau M, Mercadal-Hally M, Lera-Carballo E, García-Patos V (2020). Cutaneous manifestations in the current pandemic of coronavirus infection disease (COVID 2019). An Pediatría (English Ed.).

[16] Karaca Z, Yayli S, Çalışkan O (2020). A unilateral purpuric rash in a patient with COVID-19 infection. Dermatol Ther.

[17] Matsubara A, Sakurai M, Morita A (2021). Neutrophilic urticarial dermatosis following BNT162b2 (Pfizer–BioNTech) COVID-19 vaccination. J Cutan Immunol Allergy.

[18] Toppe E, Haas N, Henz BM (1998). Neutrophilic urticaria: clinical features, histological changes and possible mechanisms. Br J Dermatol.

[19] Wu M-L, Liu F-L, Sun J, Li X, He X-Y, Zheng H-Y (2021). SARS-CoV-2-triggered mast cell rapid degranulation induces alveolar epithelial inflammation and lung injury. Signal Transduct Target Ther.

[20] Theoharides TC (2021). Potential association of mast cells with coronavirus disease 2019. Ann Allergy Asthma Immunol.

[21] Rathore APS, Mantri CK, Aman SAB, Syenina A, Ooi J, Jagaraj CJ (2019). Dengue virus–elicited tryptase induces endothelial permeability and shock. J Clin Invest.

[22] Muntean IA, Pintea I, Bocsan IC, Dobrican CT, Deleanu D (2021). COVID-19 disease leading to chronic spontaneous urticaria exacerbation: a Romanian retrospective study. Healthcare (Basel Switzerland)..

[23] Maemura T, Kuroda M, Armbrust T, Yamayoshi S, Halfmann PJ, Kawaoka Y (2021). Antibody-dependent enhancement of SARS-CoV-2 infection is mediated by the IgG receptors FcγRIIA and FcγRIIIA but does not contribute to aberrant cytokine production by macrophages. MBio.

[24] Johansson SGO. The discovery of immunoglobulin E and its role in allergy. In 2014. p. 150–4. https://www.karger.com/Article/FullText/358621. 10.1159/000358621 24925395

[25] Ricke D, Gherlone N, Fremont-Smith P, Tisdall P, Fremont-Smith M (2020). Kawasaki disease, multisystem inflammatory syndrome in children: antibody-induced mast cell activation hypothesis. J Pediatr Pediatr Med..

[26] Eliakim-Raz N, Leibovici-Weisman Y, Stemmer A, Ness A, Awwad M, Ghantous N (2021). Antibody titers before and after a third dose of the SARS-CoV-2 BNT162b2 vaccine in adults aged ≥60 years. JAMA.

[27] Salvagno GL, Henry BM, di Piazza G, Pighi L, de Nitto S, Bragantini D (2021). Anti-spike S1 IgA, anti-spike trimeric IgG, and anti-spike RBD IgG response after BNT162b2 COVID-19 mRNA vaccination in healthcare workers. J Med Biochem.

[28] Zhao Y, Qin L, Zhang P, Li K, Liang L, Sun J (2020). Longitudinal COVID-19 profiling associates IL-1RA and IL-10 with disease severity and RANTES with mild disease. JCI Insight..

[29] Zhan Y, Zou S, Hua F, Li F, Ji L, Wang W (2014). High-dose dexamethasone modulates serum cytokine profile in patients with primary immune thrombocytopenia. Immunol Lett.

[30] Islam F, Dhawan M, Nafady MH, Bin ET, Mitra S, Choudhary OP (2022). Understanding the omicron variant (B11529) of SARS-CoV-2: mutational impacts, concerns, and the possible solutions. Ann Med Surg..

[31] Gagnier JJ, Kienle G, Altman DG, Moher D, Sox H, Riley D (2014). The CARE guidelines: consensus-based clinical case report guideline development. J Clin Epidemiol.

